# Fgf9 inhibition of meiotic differentiation in spermatogonia is mediated by Erk-dependent activation of Nodal-Smad2/3 signaling and is antagonized by Kit Ligand

**DOI:** 10.1038/cddis.2015.56

**Published:** 2015-03-12

**Authors:** V Tassinari, F Campolo, V Cesarini, F Todaro, S Dolci, P Rossi

**Affiliations:** 1 Dipartimento di Biomedicina e Prevenzione, Università degli Studi di Roma Tor Vergata, Rome, Italy

## Abstract

Both fibroblast growth factor 9 (Fgf9) and Kit Ligand (Kl) signal through tyrosine kinase receptors, yet they exert opposite effects on meiotic differentiation in postnatal spermatogonia, Fgf9 acting as a meiosis-inhibiting substance and Kl acting as a promoter of the differentiation process. To understand the molecular mechanisms that might underlie this difference, we tried to dissect the intracellular signaling elicited by these two growth factors. We found that both Fgf9 and Kl stimulate Erk1/2 activation in Kit+ (differentiating) spermatogonia, even though with different time courses, whereas Kl, but not Fgf9, elicits activation of the Pi3k-Akt pathway. Sustained Erk1/2 activity promoted by Fgf9 is required for induction of the autocrine Cripto-Nodal-Smad2/3 signaling loop in these cells. Nodal signaling, in turn, is essential to mediate Fgf9 suppression of the meiotic program, including inhibition of Stra8 and Scp3 expression and induction of the meiotic gatekeeper Nanos2. On the contrary, sustained activation of the Pi3k-Akt pathway is required for the induction of Stra8 expression elicited by Kl and retinoic acid. Moreover, we found that Kl treatment impairs Nodal mRNA expression and Fgf9-mediated Nanos2 induction, reinforcing the antagonistic effect of these two growth factors on the meiotic fate of male germ cells.

In the mouse testis, competence to enter meiosis is acquired during the differentiative stages in which spermatogonia undergo Kit-dependent mitotic divisions, but not in Kit-negative spermatogonial stem cells. Retinoic acid (RA) stimulates Kit expression in spermatogonia and Kit Ligand (Kl) expression in Sertoli cells.^1^ Kl is essential to promote the mitotic expansion of Kit+ premeiotic germ cells.^2^ However, the concerted action of both RA and Kl can induce meiotic entry of *in vitro* cultured Kit+ spermatogonia.^1^ Indeed, both RA and Kl increase the expression of genes that are fundamental for the beginning of the meiotic process,^1, 3^ and in particular of Stimulated by Retinoic Acid Gene 8 (Stra8), which is essential for the switch from the mitotic cell cycle to the meiotic program.^1^ Selective inhibitors of Kit tyrosine-kinase activity block both RA- and Kl-induced meiotic entry, suggesting that the two factors converge on common, Kit-dependent, signaling pathways.^1^ RA- and Kl-induced meiotic entry is mediated, at least in part, by activation of the Pi3k-Akt pathway. RA promotes meiosis also by inhibiting the expression of the meiotic gatekeeper Nanos2, an RNA-binding protein that silences genes essential for spermatogonial differentiation and meiotic entry.^4^ Fibroblast growth factor 9 (Fgf9) secreted by Sertoli cells acts as a meiosis-inhibiting substance by increasing Nanos2 levels in premeiotic spermatogonia, thus opposing the RA/Kl/Kit axis.^4^ In the male fetal gonad, meiotic entry of male fetal gonocytes is inhibited by the action of Cyp26b1, which degrades RA of mesonephric origin.^5, 6^ Fgf9 contributes to prevent meiosis in the male fetal gonad by inducing Nanos2 expression in gonocytes.^4, 7^ Several recent works have shown that Nodal (a Tgf*β* superfamily member) plays an important role in the inhibition of the meiotic program of fetal male germ cells.^8, 9, 10^ Moreover, it has been demonstrated that the Fgf9 inhibitory effect on meiotic entry of fetal gonocytes might be mediated, at least in part, by activation of Cripto-Nodal signaling.^9^

In the present work, we show that the Fgf9 antimeiotic effect is mediated by activation of the Cripto-Nodal-Smad2/3 signaling in postnatal spermatogonia, and that this activation requires Fgf9-dependent stimulation of the Erk1/2 pathway. On the contrary, Kl promotes meiotic differentiation through the activation of the Pi3k-Akt pathway and inhibition of Nodal expression.

## Results

### Fgf receptors expression in postnatal spermatogonia

**Figure 1:**
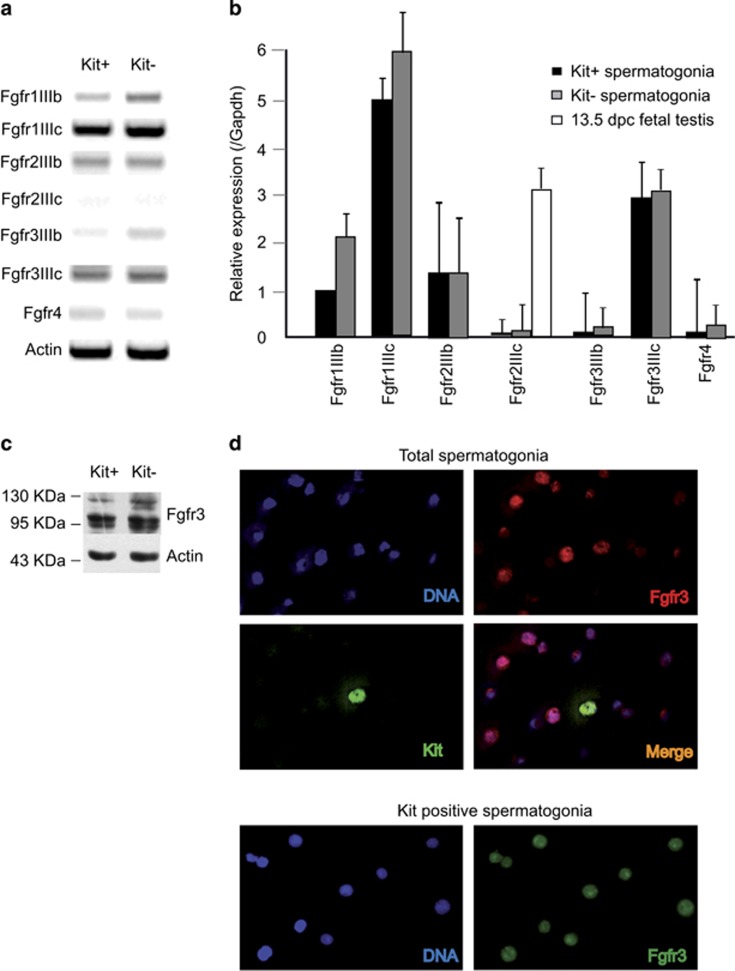
Fgfrs expression in postnatal mouse spermatogonia. (**a**) Semiquantitative RT-PCR analysis for mRNA expression of different Fgfr isoforms in Kit+ and Kit- spermatogonia. (**b**) Quantitative RT-PCR analysis for mRNA expression of different Fgfr isoforms in Kit+ and Kit- spermatogonia. Fgf2IIIC mRNA expression was also evaluated in 13.5 dpc fetal male gonads. Data represent the mean±S.D. from three independent experiments. (**c**) Western blot analysis of Fgfr3 expression in in Kit+ and Kit- spermatogonia. (**d**) Immunofluorescence analysis of Fgfr3 expression in the total population of isolated spermatogonia from 6 dpp mice (top panels), and in purified Kit+ spermatogonia from 7 dpp mice (bottom panels). In the top panels, a Kit+ spermatogonium can be identified in the population of mostly undifferentiated germ cells through EGFP fluorescence, as these cells were derived from testes of transgenic mice in which EGFP expression is driven by Kit transcription regulatory sequences^1, 16^

We previously showed that Fgf9 negatively regulates meiotic entry of postnatal and fetal male germ cells.^4^ To understand which of the Fgf receptors was mediating this effect, we first performed an RT-PCR analysis for the different Fgfr isoforms in purified Kit-positive (Kit+, differentiating) and Kit-negative (Kit-, undifferentiated) spermatogonia. As shown in Figures 1a and b and Supplementary Figure 2A, we found that the predominant Fgfr isoform transcripts expressed in spermatogonia were Fgfr1-IIIc and Fgfr3-IIIc, the latter being a high-affinity receptor for Fgf9.^11^ Fgfr2, which has been reported to be expressed in Sertoli cells,^12^ was barely detectable in spermatogonia. In particular, Fgf2IIIC was completely absent both in Kit- and Kit+ spermatogonia (Figures 1a and b), even though it was highly expressed in fetal male gonads (Figure 1b). By western blot, we confirmed that both Kit- and Kit+ spermatogonia expressed Fgfr3 (Figure 1c). We found two bands: a major band of approximately 95 KDa and a minor band of approximately 120 KDa (probably representing the glycosylated form). To verify the specificity of the anti-Fgfr3 antibody, we performed RNA interference on the T98G human glioblastoma cell line, which expresses FGFR3. We chose this cell line as it was not possible to induce RNA interference in primary spermatogonia, because of the very low efficiency of RNA transfection in these cells. As shown in Supplementary Figure 2B, RNA interference in T98G cells efficiently downregulated FGFR3 bands with sizes identical to those found in primary spermatogonia, demonstrating specificity of the antibody. By immunofluorescence analysis (Figure 1d), we also found that while spermatogonia were intensely stained, the contaminating somatic cells present in the total population were completely negative, (Figure 1d top panel), further confirming specificity of the antibody. Interestingly Fgfr3 positivity in spermatogonia was found not only at the membrane/cytoplasmatic level, but also in the nuclear compartment, as previously observed in human spermatogonia^13^ and in other cell types.^14^ Both localizations were not modified by incubation with Fgf9 (not shown). These results are consistent with previous immunohistochemical evidence for selective expression of Fgfr3 in mouse postnatal spermatogonia.^15^

**Figure 2:**
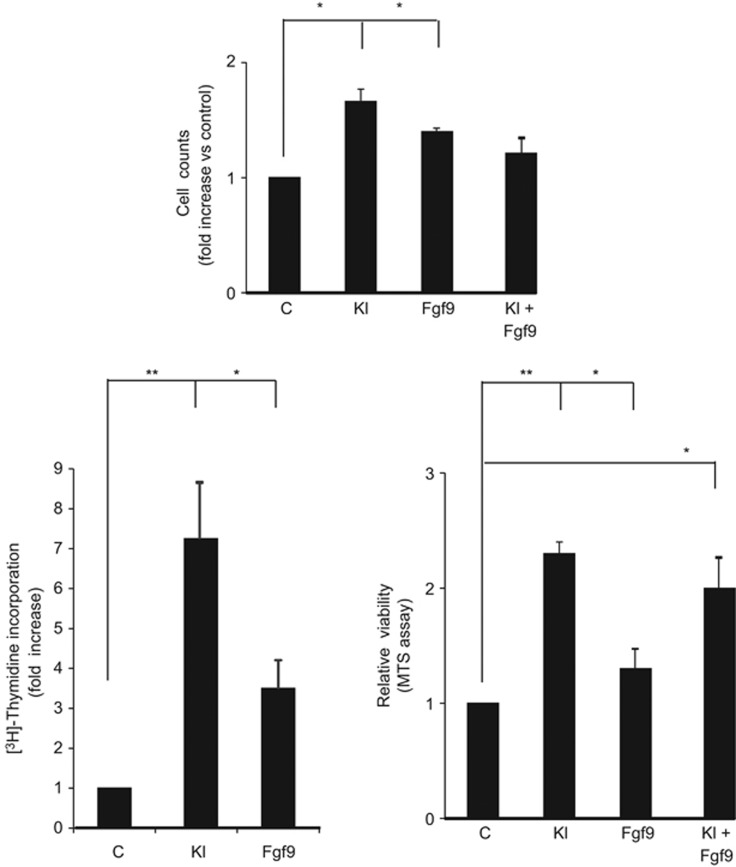
Fgf9 stimulates proliferation of spermatogonia. Top panel: cell count analysis of total spermatogonia after 24 h of culture in the presence or absence of Kl, Fgf9 or both. Bottom panels: analysis of [^3^H]-thymidine incorporation (left panel) and cell viability measured by MTS assay (right panel) of *in vitro* cultured purified Kit+ spermatogonia treated overnight with Kl, Fgf9 or both factors. Data are expressed as the average fold increase with respect to untreated cells in three independent experiments. Bars represent the mean ±S.D.

Fgfs lead primarily to cell proliferation although cell differentiation or cell migration can also be observed.^17, 18^ To understand whether Fgf9 could induce spermatogonia proliferation, we first performed cell counts of total spermatogonia after 24 h of culture in the presence or absence of Kl, Fgf9 or both (Figure 2, top panel). We found that both Kl and Fgf9 induced a significant increase of cell numbers, but they did not show any additive effect. The same results were obtained by [^3^H]-thymidine incorporation and, MTS assays on Kit+ cells, which are the cells committed to enter meiosis. As shown in Figure 2, bottom panels, an increase of DNA synthesis and cell viability were induced by Fgf9 treatment. As expected, also Kl induced an increase of both parameters, although the proliferation rate was twice that found in Fgf9-treated samples. Also in Kit+ cells, no additive effect was found when both factors were added together, suggesting that Kl and Fgf9 might converge on common pathways to signal mitogenic stimuli on Kit+ cells.

### Fgf9 activates Cripto/Nodal pathway in Kit positive spermatogonia

**Figure 3:**
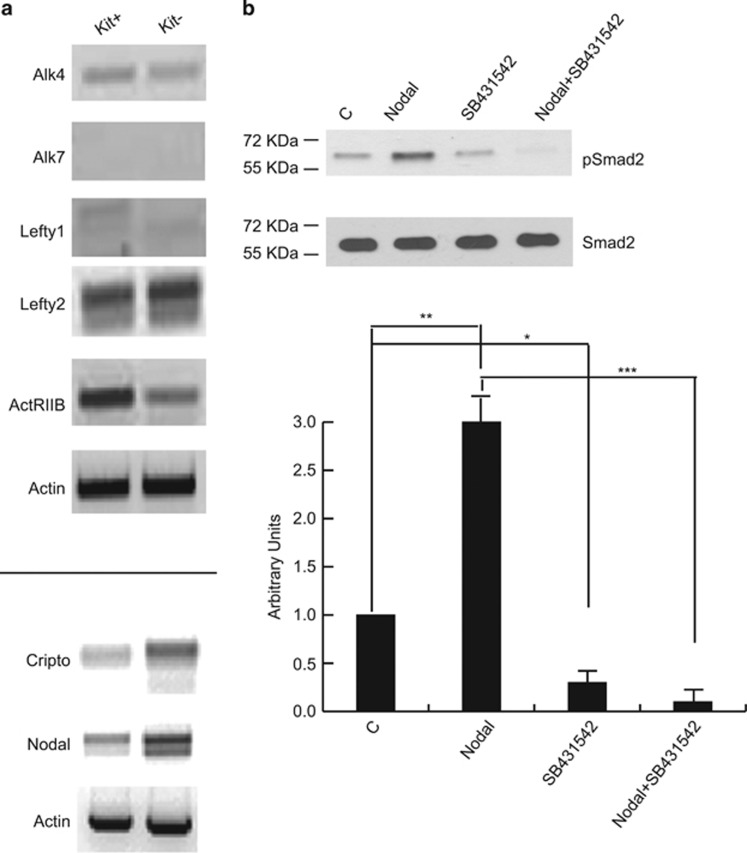
Expression and activity of the Cripto-Nodal signaling pathway in postnatal mouse spermatogonia. (**a**) Semiquantitative RT-PCR analysis of mRNA expression of the indicated elements of the Cripto-Nodal signaling pathway in Kit+ and Kit- spermatogonia. (**b**) Western blot analysis of Smad2 phosphorylation in cultured untreated Kit+ spermatogonia and in the same cells treated overnight with recombinant Nodal, in the presence or absence of the Alk4/7 selective inhibitor SB431542. Densitometric analysis of western blots from three independent experiments is shown below. Bars represent the mean ±S.D.

It has been recently reported that Nodal expression in male fetal germ cells autocrinally prevents them to enter meiosis^8^ and that Fgf9 induces Cripto, a co-receptor for Nodal, in the same cell type.^9^ To investigate whether the Cripto/Nodal system was active also in postnatal germ cells, we first analyzed the expression pattern of members of the Nodal signaling pathway. We found that Alk4, ActRIIB and Lefty2 transcripts were expressed in both Kit+ and Kit- spermatogonia, whereas Alk7 and Lefty1 were completely absent (Figure 3a). Cripto and Nodal transcripts were expressed at higher levels in Kit- spermatogonia. Following 1h incubation with 100 ng/ml Nodal, pSmad2 levels were rapidly induced in Kit+ spermatogonia and completely abolished by co-treatment with SB431542, a specific Alk4/5/7 inhibitor (Figure 3b). This result indicates that the Cripto/Nodal system can be potentially activated in premeiotic male germ cells.

**Figure 4:**
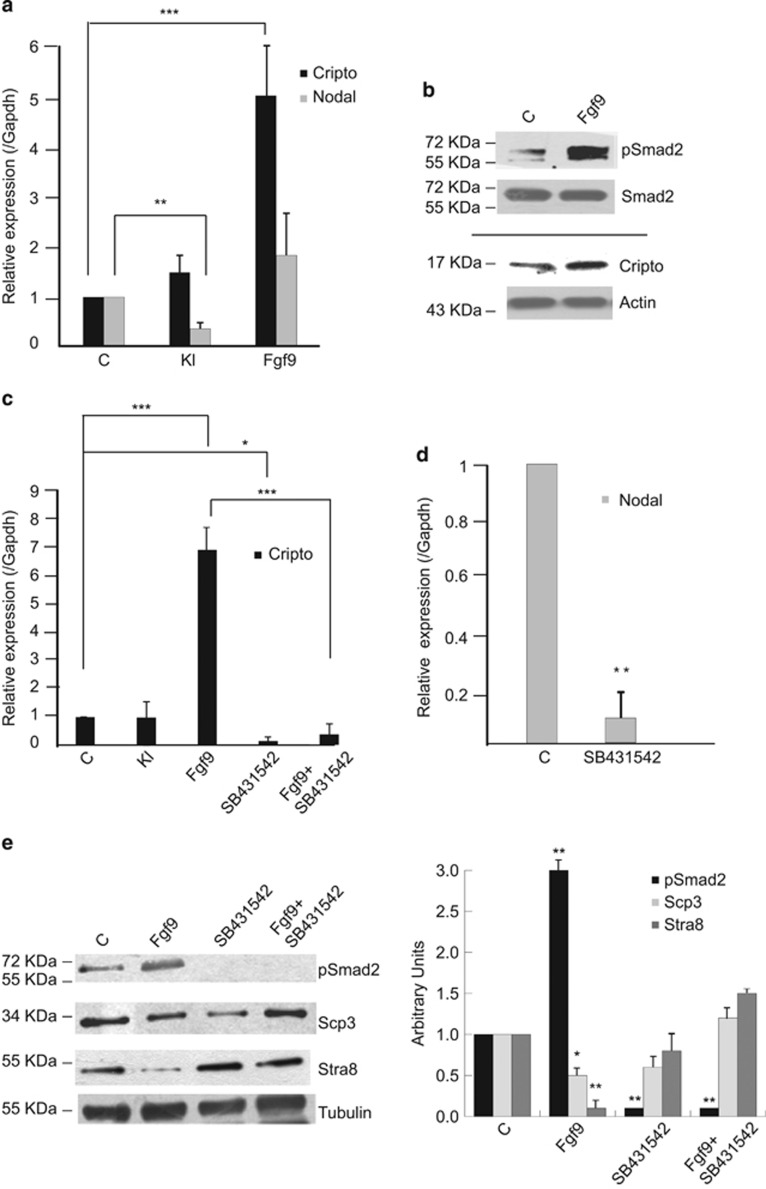
Fgf9-mediated meiotic inhibition in Kit+ spermatogonia is mediated by activation of Nodal signaling. (**a**) Quantitative RT-PCR analysis of Cripto and Nodal mRNA expression in untreated Kit+ spermatogonia and in the same cells treated overnight with Kl or Fgf9. Data represent the mean±S.D. from five independent experiments. (**b**) Representative western blot analysis of Smad2 phosphorylation and Cripto expression in Kit+ control spermatogonia and in the same cells treated overnight with Fgf9. (**c**) Quantitative RT-PCR analysis of Cripto mRNA expression in untreated Kit+ spermatogonia and in the same cells treated overnight with Kl or with Fgf9, in the presence or absence of the Alk4/7 selective inhibitor SB431542. Data represent the mean±S.D. from four independent experiments. (**d**) Quantitative RT-PCR analysis of Nodal mRNA expression in untreated Kit+ spermatogonia and in the same cells treated overnight the Alk4/7 selective inhibitor SB431542. Data represent the mean±S.D. from three independent experiments. (**e**) Western blot analysis of Smad2 phosphorylation and expression of meiotic markers (Stra8 and Scp3) in cultured untreated Kit+ spermatogonia and in the same cells treated overnight with Fgf9, in the presence or absence of the Alk4/7 selective inhibitor SB431542. Densitometric analysis of western blots from four independent experiments is shown on the right. Bars represent the mean±S.D.

As Fgf9 treatment for 24 h inhibits meiosis both in fetal and postnatal male germ cells *in vitro*,^4, 7^ we investigated whether this stimulation might influence Cripto and/or Nodal levels. To dissect the mechanisms of anti-meiotic effect of Fgf9, we focused on Kit+ spermatogonia. The peculiarity of these cells is that they can proliferate or enter the meiotic process *in vivo* and *in vitro* depending on the environmental stimuli, whereas Kit- cells are not able to enter meiosis before the onset of Kit expression.^1, 4, 19^ As shown in Figures 4a and b, Fgf9 induced a strong increase of Cripto levels both at mRNA and protein levels, together with the induction of Smad2 phosphorylation (Figure 4b), while Nodal expression was unaffected (Figure 4a). On the contrary, Kl did not enhance Cripto mRNA levels, but it inhibited Nodal expression (Figure 4a). Inhibition of Nodal signaling by SB431542 treatment completely blocked Fgf9 induction of Cripto expression (Figure 4c) and decreased endogenous Nodal mRNA levels (Figure 4d). These results demonstrate the existence of a positive Cripto/Nodal autocrine loop in postnatal spermatogonia. Activation of the Cripto/Nodal signaling pathway was required, at least in part, for mediating Fgf9 anti-meiotic effects in Kit+ spermatogonia, as suggested by the full recovery of Stra8 and Scp3 expression following the addition of SB431542 to Fgf9-treated cells (Figure 4e).

### Fgf9 activates Erk1/2, but not Akt signaling in Kit-positive spermatogonia

**Figure 5:**
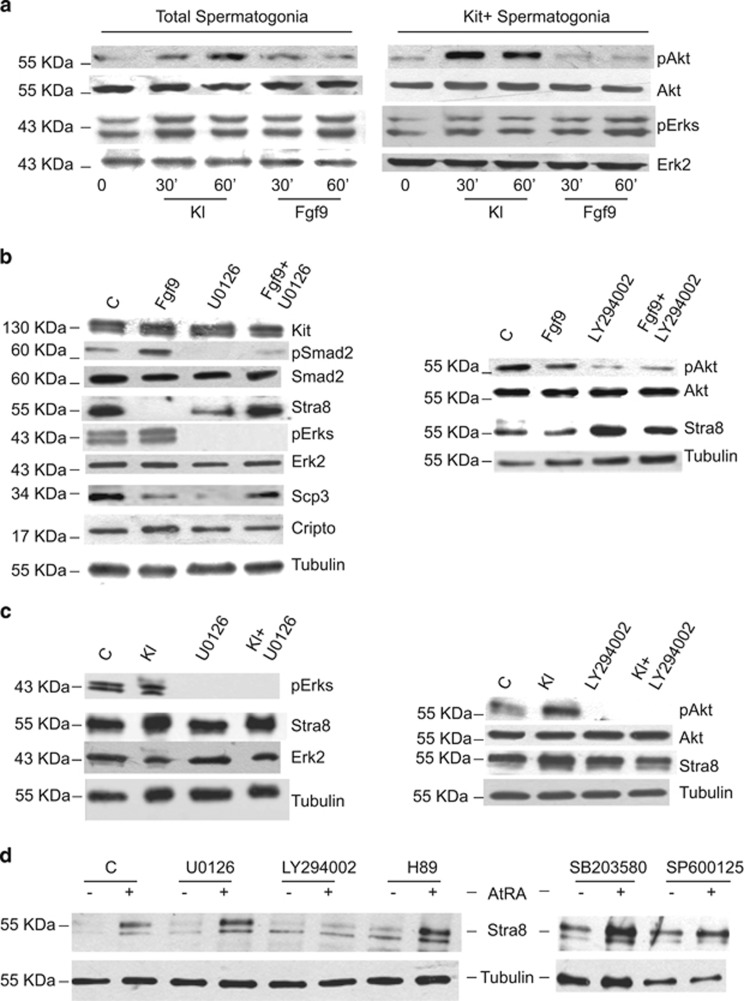
Erk1/2 signaling is required for Fgf9-mediated Cripto/Nodal activation and meiotic inhibition. (**a**) Representative western blot analysis of Erks and Akt phosphorylation in total and in purified Kit+ spermatogonia after short-term incubation with Kl or with Fgf9. These experiments were repeated more than 10 times with similar results. (**b**) Left panel: Western blot analysis of Smad2 and Erk phosphorylation and expression of Cripto and of meiotic markers (Stra8 and Scp3) in cultured untreated Kit+ spermatogonia and in the same cells treated overnight with Fgf9, in the presence or absence of the Mek selective inhibitor U0126. Right panel: Western blot analysis of Akt phosphorylation and Stra8 expression in cultured untreated Kit+ spermatogonia and in the same cells treated overnight with Fgf9, in the presence or absence of the Pi3k selective inhibitor LY294002. (**c**) Left panel: Western blot analysis of Erks phosphorylation and Stra8 expression in cultured untreated Kit+ spermatogonia and in the same cells treated overnight with Kl, in the presence or absence of the Mek selective inhibitor U0126. Right panel: Western blot analysis of Akt phosphorylation and Stra8 expression in cultured untreated Kit+ spermatogonia and in the same cells treated overnight with Kl, in the presence or absence of the Pi3k selective inhibitor LY29400. (**d**) Western blot analysis of Stra8 expression in cultured untreated Kit+ spermatogonia (-) and in the same cells treated overnight with all-trans Retinoic acid (AtRA) (+) in the absence or the presence of the indicated selective signaling inhibitors (U0126 for Mek; LY294002 for Pi3k; H89 for pkA; SB203580 for p38; SP600125 for Jnk). In these experiments, the Stra8 signal appears as a doublet rather than a single band, as protein extracts were separated by SDS-PAGE in 4–20% gradient gels instead than in uniform gels

Both Fgf9 and Kl signal through tyrosine kinase receptors, yet they exert opposite effects on meiotic differentiation in postnatal spermatogonia. To understand the molecular mechanisms that might underlie these differences, we tried to dissect the intracellular signaling elicited by these growth factors. To this end, we studied Mek, Pi3k Jnk, Plc*γ* and p38 activation pathways in spermatogonia by monitoring the levels of phosphoErk1/2, phospho-Akt, phospho-Jnk, phospho-Plc*γ* and phospho-p38, respectively, in time course experiments. Jnk, Plc*γ* and p38 pathways were not found to be significantly influenced neither by Fgf9 nor by Kl treatments (data not shown). However, we found that Fgf9 increased phospho Erk1/2 levels between 30 and 60 min of stimulation, and this effect was evident both in total spermatogonia and in Kit+ cells (Figure 5a). Overnight stimulation with Fgf9 still resulted in increased pErk1/2 levels with respect to control cells (Figure 5b). Kl stimulation, on the contrary, induced a transient Erk1/2 activation after 30 min and then it decreased to the control levels within 1 h (Figure 5a). We did not find any increase of phospho-Akt levels following either transient or overnight Fgf9 stimulation (Figure 5a and b), whereas Kl induced a rapid and persistent Akt phosphorylation (Figure 5a), which was evident also after overnight stimulation (Figure 5c).

### Erk1/2 signaling is required for Fgf9-mediated Cripto/Nodal activation and meiotic inhibition

To understand whetehr Cripto/Nodal stimulation induced by Fgf9 requires Erk1/2 activation, spermatogonia cultures were exposed to Fgf9 in the presence or absence of U0126, a specific Mek inhibitor. As shown in Figure 5b (left panel), Fgf9-mediated induction of both pSmad2 and Cripto levels were completely abolished by U0126 treatment, whereas LY294002 did not exert any effect (Figure 5b, right panel). Similarly, Fgf9-mediated inhibition of Stra8 and Scp3 expression was completely reverted in the presence of the Mek inhibitor (Figure 5b, left panel). We found that Kl induced Stra8 expression and this effect was completely abolished by the treatment with LY294002, a specific inhibitor of the Pi3k signaling pathway, but not by U0126 (Figure 5c). In line with this result, we found that Pi3k signaling was specifically required also for RA-mediated induction of Stra8 expression, whereas inhibition of Mek, PKA, p38 or Jnk pathways showed no effect (Figure 5d). These results confirm previously reported studies showing the importance of Pi3k/Akt signaling pathway in the pro-meiotic effect of Kl and RA on cultured spermatogonia.^1^

### Fgf9-induced increase of Nanos2 expression depends on Nodal signaling and is inhibited by Kl

**Figure 6:**
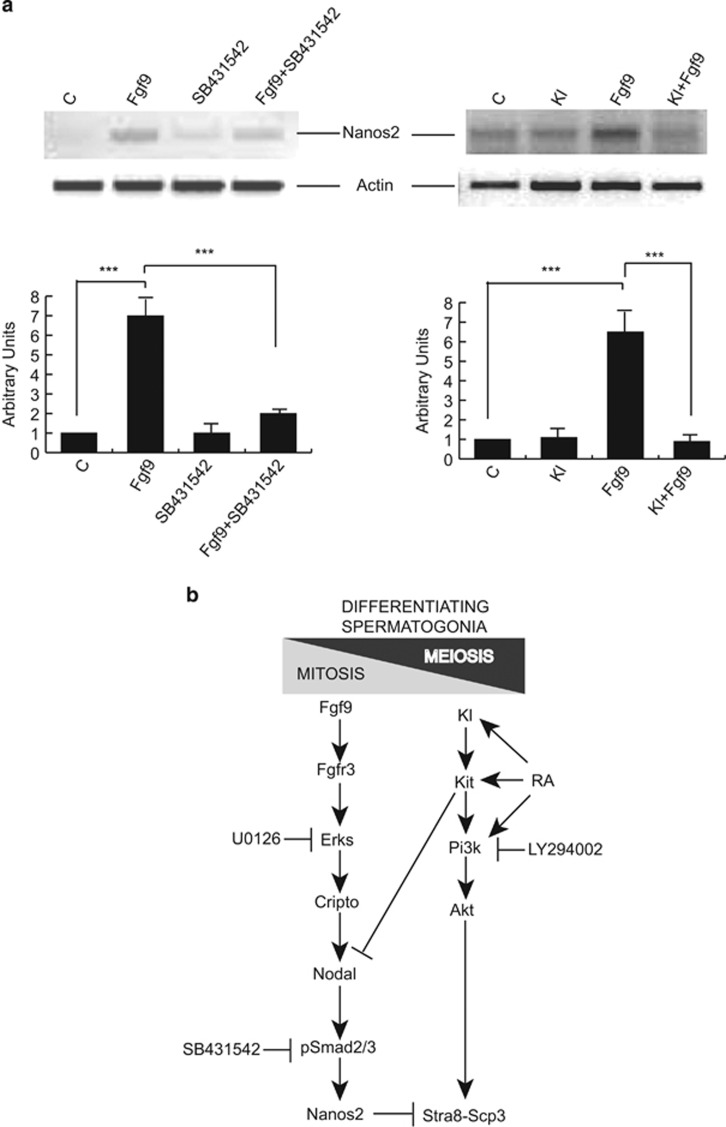
Fgf9-induced increase of Nanos2 expression depends on Nodal signaling and is inhibited by Kl. (**a**) Left panel: Semiquantitative RT-PCR analysis of mRNA expression of Nanos2 in untreated Kit+ spermatogonia and in the same cells cultured overnight with Fgf9, in the presence or absence of the Alk4/7 selective inhibitor SB431542. In this experiment, we used Nanos2 primers '1' listed in Supplementary Table 1. Right panel: Semiquantitative RT-PCR analysis of mRNA expression of Nanos2 in untreated Kit+ spermatogonia and in the same cells cultured overnight with Kl, with Fgf9 or with both growth factors. In this experiment, we used Nanos2 primers ‘2' listed in Supplementary Table 1. For both panels, the results of densitometric analysis from three independent experiments is shown below. Bars represent the mean ±S.D. (**b**) Schematic representation which summarizes the main findings of our present work: Fgf9 maintains differentiating (Kit+) spermatogonia in the mitotic condition and prevent meiosis (Stra8 and Scp3 expression) through a cascade involving sustained Erks activation and the consequent stimulation of the Cripto-Nodal-pSmad2/3 signaling, which leads to expression of the meiotic gatekeeper Nanos2. On the opposite, retinoic acid (RA) and Kl/Kit signaling inhibit Nodal expression and stimulate Pi3k-Akt signaling, which is required for Stra8 and Scp3 expression, with the consequent mitotic-meiotic switch

One of the downstream effectors of Fgf9 anti-meiotic activity is the upregulation of the RNA-binding protein Nanos2.^4, 7^ We confirmed that Fgf9 treatment increases Nanos2 expression in Kit+ spermatogonia, and found that this effect also required Nodal activity, as it was abolished by SB431542 treatment (Figure 6a, left panel and Supplementary Figure 3A). Interestingly, Kl co-treatment strongly decreased the Fgf9-mediated induction of Nanos2 mRNA levels (Figure 6a, right panel and Supplementary Figure 3B).

## Discussion

In this paper, we show that Kl and Fgf9, both stimulating receptor tyrosine kinase activities (and thus presumably sharing common signal transduction pathways), act on the same germ cell type (Kit+ spermatogonia), yet they exert opposite effects (promotion *versus* prevention of meiotic differentiation). We aimed to dissect the differences in the signaling pathways activated by these two antagonistic growth factors and to identify the events that lead to meiotic entry (Kl) or meiotic inhibition (Fgf9) in differentiating spermatogonia. Firstly, we identified Fgfr3 as the most abundant potential transducer of Fgf9 signaling in spermatogonia. Both Fgf9 and Kl stimulate Erk1/2 activation in spermatogonia, even though with different time courses, whereas Kl, but not Fgf9, elicits activation of the Pi3k-Akt pathway.

We have previously shown that a transient Erk1/2 activation, as well as Pi3k-Akt signaling, were both essential for the mitogenic effect exerted by Kl on differentiating spermatogonia.^2^ We now show that sustained activation of Erk signaling by Fgf9 also elicits a mitogenic effect on these cells, even though this effect is less evident, presumably because it is exerted in a subset of Kit+ cells. Contrary to what reported in the human postnatal testis,^9^ we found that members of the Nodal pathway are expressed in both Kit- and Kit+ mouse spermatogonia, at least in the pre-puberal testis, during the first wave of spermatogenesis. More importantly, we found that Fgf9 is required for activation of the autocrine Cripto-Nodal-Smad2/3 signaling loop in these cells, as previously reported in fetal gonocytes.^8, 9, 10^ Nodal signaling appears to be essential in mediating Fgf9 suppression of the meiotic program, including inhibition of Stra8 and Scp3 and induction of Nanos2 expression. Stimulation of the Nodal autocrine loop requires the sustained Erk1/2 activation triggered by Fgf9. On the contrary, sustained activation of the Pi3k-Akt pathway is required for the induction of Stra8 expression elicited by both Kl and RA.

In agreement with the importance of Kl-mediated activation of the Pi3k-Akt pathway in promoting the meiotic program, a point mutation in the Kit gene, which impairs its interaction with the Pi3k-Akt signaling pathway, causes male sterility owing to a block in the initial stages of spermatogenesis, soon after the beginning of Kit expression.^20, 21^ Furthermore, male mice expressing a catalytically inactive p110*β* (the catalytic subunit of Pi3k) develop testicular hypotrophy and impaired spermatogenesis, leading to a phenotype of oligo-azoospermia and defective fertility.^22^

We also found that Kl treatment of Kit+ spermatogonia reduces Nodal mRNA expression; however, this effect was not apparently mediated by activation of the Pi3k-Akt pathway (data not shown). Kl exerted a negative effect also on Fgf9-mediated Nanos2 induction, reinforcing the antagonistic effect of these two growth factors on the meiotic fate of male germ cells.

Selective activation of the Erk1/2-Nodal axis by Fgf9 and of the Pi3k-Akt axis by Kl might not be the only important difference between the action of the Fgfr and Kit tyrosine kinase receptors in postnatal male germ cells. Another important differential feature is the nuclear compartmentalization of Fgfr3 in postnatal spermatogonia, whereas Kit is exclusively expressed on the cell membrane. However, involvement of nuclear translocation of Fgfr3 induced by Fgf9 is an unlikely mechanism for the control of Nodal signaling, as we did not observe any evident difference in Fgfr3 nuclear localization in Fgf9-treated spermatogonia compared with the control.

Overall, our present results suggest that fine tuning of the autocrine Cripto-Nodal-Smad2/3 signaling loop is crucial for the decision of differentiating spermatogonia to undergo the meiotic program. Fgf9 stimulates the loop in an Erk1/2-dependent manner, leading to inhibition of meiotic entry. The RA/Kl system acts in the opposite way by promoting Stra8 expression through stimulation of the Pi3k-Akt pathway (which is not affected by Fgf9) and by parallel inhibition of Nodal signaling and Nanos2 expression (Figure 6B).

## Materials and Methods

### Cell isolation and culture

Primary postnatal male germ cells from testes of 6–7 days postpartum (dpp) CD1 albino mice were obtained by sequential enzymatic digestion, as previously reported.^1^ Total germ cells were pre-plated in modified Eagle's medium (Gibco, Milan, Italy) with 20 mM glutamine (Gibco), 2 mM pyruvic acid (Sigma Aldrich, Milan, Italy), 1 mM lactic acid (Sigma Aldrich), non-essential amino acids (Gibco), 100 U/ml penicillin and 100 *μ*g/ml streptomycin (Gibco) supplemented with 10% serum (FBS) to allow most of the somatic cells to adhere to the plastic dishes. After pre-plating, cells were separated by magnetic-activated cell sorting to obtain Kit-positive and Kit-negative spermatogonia using CD117 conjugated, microbeads (Miltenyi Biotech, Bergisch-Gladbach, Germany), as previously described.^23^ Purity of both Kit-positive and Kit-negative fractions was checked by western blot and RT-PCR analysis for Oct4, Plzf, Sohlh1 and Kit, as previously described.^23^ Purity of the Kit-positive cells after immunomagnetic cell sorting was also checked by exploiting transgenic mice in which EGFP expression is driven by transcriptional *Kit* regulatory regions.^16^ As shown in Supplementary Figure 1A cell purity was estimated to be more than 95%. This result was also confirmed by Kit immunostaining after cell sorting (Supplementary Figure 1B). Fetal gonads were isolated from male embryos at 13.5 days post coitum (dpc). Fgf9 and Kl (from Società Italiana Chimici) were dissolved in PBS 1 mg/ml BSA and used at a final concentration of 25 ng/ml and 100 ng/ml, respectively. These concentrations were chosen on the basis of dose–response experiments performed in primary spermatogonia, as previously reported.^4, 24^ All-trans-retinoic-acid (AtRA, Sigma Aldrich) was dissolved in ethanol and used at a final concentration 0.3 *μ*M.^1^ Nodal (from R&D Systems, Minneapolis, MN, USA) was dissolved in 1 mM HCl at a final concentration of 100 ng/ml. SB431542 was dissolved in DMSO and used at a final concentration 20 *μ*M. This concentration was used on the basis of previous studies performed in fetal male germ cells.^8^ The MAPK inhibitor (U0126), PI3K inhibitor (LY294002) and all the others inhibitors (H89, SB202190, SP600125) were from Calbiochem (Darmstadt, Germany) and used at 10 *μ*M concentration on the basis of previously reported experiments on germ cells.^1, 2, 8, 25^ For cell proliferation analysis, spermatogonia were incubated in the presence or absence of Kl, Fgf9 or both and cell counts were obtained after 24 h of culture.

### RNA interference

For *FGFR3* RNA interference, human gliobastoma T98G cells were plated to 80% confluence and transfected with Lipofectamine 3000 (Life Technologies, Monza, Italy), according to the manufacturer's instructions. To achieve FGFR3 knockdown, different concentration of *FGFR3* siRNA (Life Technologies Silencer Select, cat. No. 4390771) were tested (5 nM, 10 nM, 50 nM, 100 nM). Transfection was blocked after 48 h and *FGFR3* silencing was verified through western blot. The sequence of the siRNA used was 5′-CGUAAGAAGUGUUAAGUCUtt-3′.

### RT and real-time quantitative PCR

Total RNA from germ cells was extracted with Trizol reagent and treated with DNase I to avoid potential contamination by genomic DNA. DNA-free RNA was reverse-transcribed using Bioline (Taunton, MA, USA; BIO-65043) tetro cDNA Synthesis Kit according to the manufacturer's instructions.

For semiquantitative RT-PCR, 25 cycles were performed for the amplification of Actin, 38 cycles for Cripto, Nodal and all others genes amplification from male germ cells. Densitometric analyses were performed by ImageJ (http://rsb.info.nih.gov/ij/index.html).

The Applied Biosystems (Foster City, CA, USA) 7300 Real-time PCR System and SsoAdvanced Universal SYBR Green Supermix (BIO-RAD 172-5271, Hercules, CA, USA) were used for quantitative RT-PCR (RT-qPCR). The comparative 2(-Delta Delta C(T)) method was used to determine the relative quantities of mRNA, using Gapdh mRNA as the endogenous normalizer. Sequences of oligonucleotides used for RT-PCR and RT-qPCR are given in Supplementary Table 1.

### Western blotting

For western blot analysis, germ cells were harvested and washed three times with ice-cold PBS. Cell lysis was performed with 150 mM NaCl, 50 mM Tris HCl, 1% NP-40, 0.1% SDS, 0.5% sodium deoxycholate, 0.5M DTT, 0.5M NaF, 100 mM PMSF, 1M beta-glycerophosphate, 0.5 M sodium orthovanadate and protease inhibitors. Proteins were separated by SDS-PAGE in 4–20% gradient gels (Bio-Rad) or in uniform gels of 10% polyacrylamide and transferred to PVDF membrane (Amersham, Piscataway, NJ, USA). The membrane was blocked in TBS-5% BSA for 1 h at room temperature. Incubation with primary antibodies was carried out at 4 °C o/n in TBST-5% BSA and then with the appropriate horseradish peroxidase-conjugated secondary antibody (Santa Cruz, Heidelberg, Germany). Anti-FGFR3 rabbit polyclonal (Santa Cruz, sc-123) was diluted 1 : 500, Anti-Actin rabbit polyclonal (A2066, Sigma Aldrich) was diluted 1 : 2000, anti-phosho-Smad2 (Ser465/467) rabbit monoclonal was diluted 1 : 500 (Cell Signaling 138D4, Danvers, MA, USA), anti-Smad2 rabbit polyclonal (GTX111131) was from GeneTex (Irvine, CA, USA) and diluted 1 : 500, anti-Cripto rabbit polyclonal was fom Abcam (Cambridge, UK) and diluted 1 : 500 (ab139725), anti-Scp3 mouse monoclonal was diluted 1 : 1000 (Santa Cruz, sc-74569), anti-Stra8 rabbit polyclonal was diluted 1 : 1000 (ab49405), anti-Tubulin mouse polyclonal was diluted 1 : 2000 (Sigma Aldrich, T9026), anti-p-Akt (Ser 473) rabbit polyclonal was diluted 1 : 1000 (Santa Cruz, sc-7985), anti-p-Erks (Thr202/Tyr204) rabbit polyclonal was diluted 1 : 1000 (Cell Signaling 9101 S), anti-Erk2 rabbit polyclonal was diluted 1 : 1000 (Santa Cruz, sc-154), anti-Akt mouse monoclonal was diluited 1 : 1000 (Santa Cruz, sc-5298), anti-Kit rabbit polyclonal was produced by ProteoGenix (Schiltigheim, France) as previously described and^26^ diluted 1 : 1000. The complete list of antibody usage is available in Supplementary Table 2. The horseradish peroxidase conjugate was detected by chemioluminescence with an ECL kit (Bio-Rad) and auto-fluorography. Densitometric analyses were performed by ImageJ (http://rsb.info.nih.gov/ij/index.html).

### Immunofluorescence

Spermatogonia from of transgenic mice testes in which EGFP expression is driven by Kit transcription regulatory sequences^1, 16^ were adhered onto poly-L-lysine glass slides and fixed for 10 min at room temperature in 4% paraformaldehyde. Cells were washed twice in PBS, permeabilized for 10 min with PBS containing 0.1% Triton-X-100 and incubated for 1 h with PBS containing 0.5% BSA. Spermatogonia were then incubated o/n at 4 °C with anti-FGFR3 antibody which was diluted 1 : 200 and after washing, secondary antibody (Cy3-conjugated donkey anti-rabbit IgG, from Millipore, Billerica, MA, USA) was added for 1 h at room temperature. Anti-CD117-PE (Miltenyi Biotech) was used for Kit immunoflorescence. Slides were washed and mounted in 50% glycerol in PBS. Hoechst 33342 (Sigma Aldrich) was used to counterstain nuclei. Deconvolution and microscopy inspections were performed on a Leica (Milan, Italy) CTR6000 microscope.

### [^3^H]Thymidine incorporation and MTS assay

DNA synthesis was studied by [^3^H]thymidine incorporation followed by autoradiography as previously described.^24^ In these experiments, Kl and FGF9 were added o/n while incubation with [^3^H]thymidine was performed during the last 4 h of the culture.

MTS assay was performed using the CellTiter 96 AQueous One Solution Cell Proliferation Assay (Promega, Milan, Italy) according with the manufacturer's instructions. Cells were plated in a 96-well plate in 100 *μ*l of culture medium and treated with Kl or FGF9. Following an o/n incubation with the growth factors 20 ul of CellTiter 96 AQueous One Solution Reagent were added, incubated for 4 h and then recorded the absorbance at 490 nm with a 96-well plate reader.

### Statistical analysis

Statistical comparisons between control and treatments were determined by ANOVA test. The Student's *t* test was used to assess the significance if two comparisons were planned. All experiments were performed at least three times. Values are reported as mean and S.D. Asterisks indicate the level of statistical significance (*, *P*<0.05; **, *P*<0.001; ***,*P*<0.0001).

## Abbreviations

Fgf9: Fibroblast growth factor 9
Fgfr: Fgf receptor
Erks: Extracellular-signal-regulated kinases
Pi3k: Phosphatidylinositol-4,5-bisphosphate 3-kinase
Akt: v-akt murine thymoma viral oncogene homolog
Cripto: teratocarcinoma-derived growth factor 1
Tgf*β*: transforming growth factor beta
Smad: Small mothers against decapentaplegic
Nanos2: Nanos homolog 2
EGFP: Enhanced green fluorescent protein
RA: Retinoic acid
Kl: Kit ligand
Stra8: Stimulated by retinoic acid gene 8
Scp3: Synaptonemal complex protein 3
