# Supplementary figures and images for: Fgf9 inhibition of meiotic differentiation in spermatogonia is mediated by Erk-dependent activation of Nodal-Smad2/3 signaling and is antagonized by Kit Ligand
Source: Cell Death Dis. 2015 Mar 12;6(3):e1688–. doi: 10.1038/cddis.2015.56 (PMC4385934; doi:10.1038/cddis.2015.56)

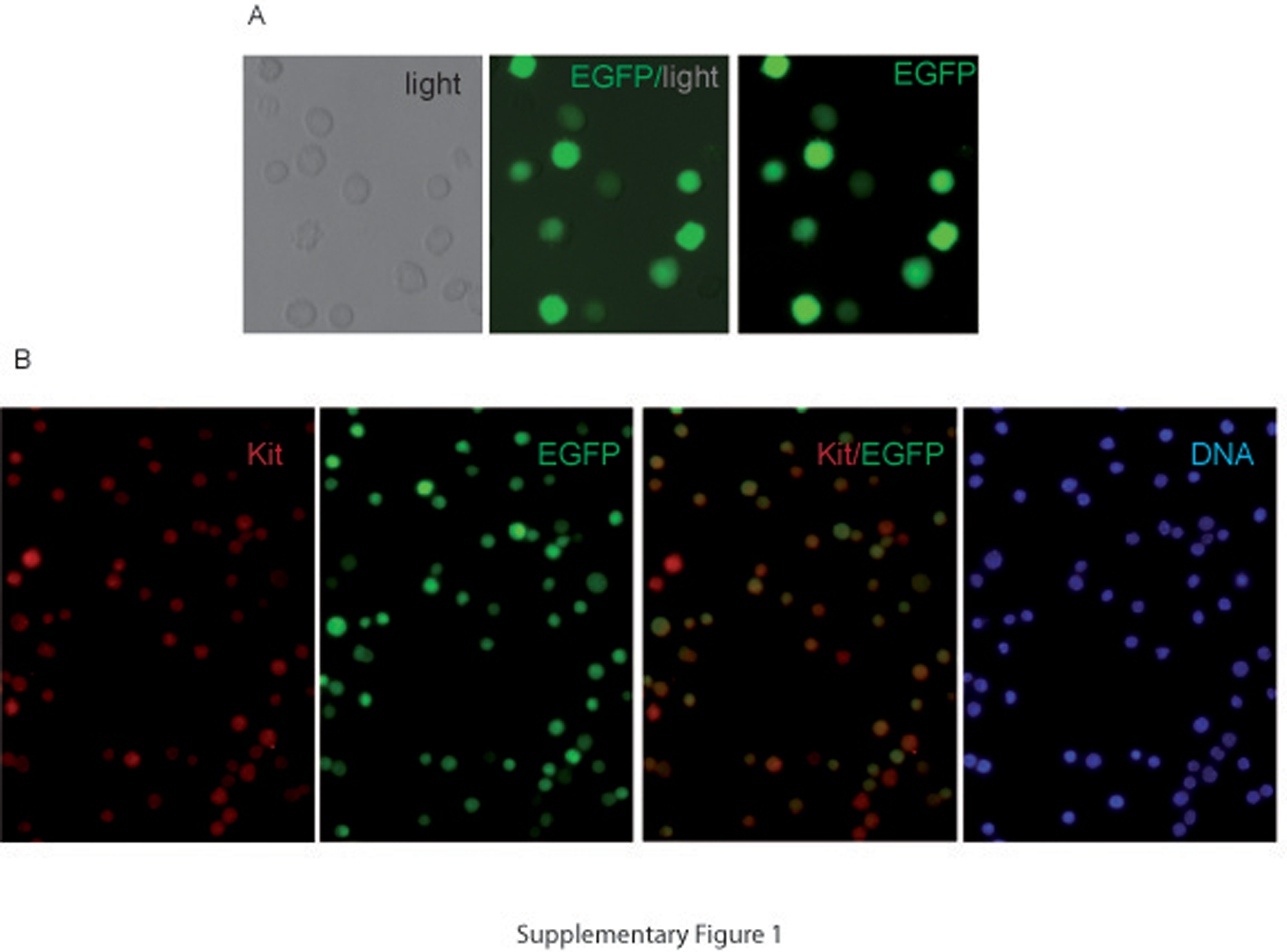

Supplement: Supplementary Figure 1 [file cddis201556x1.tif]

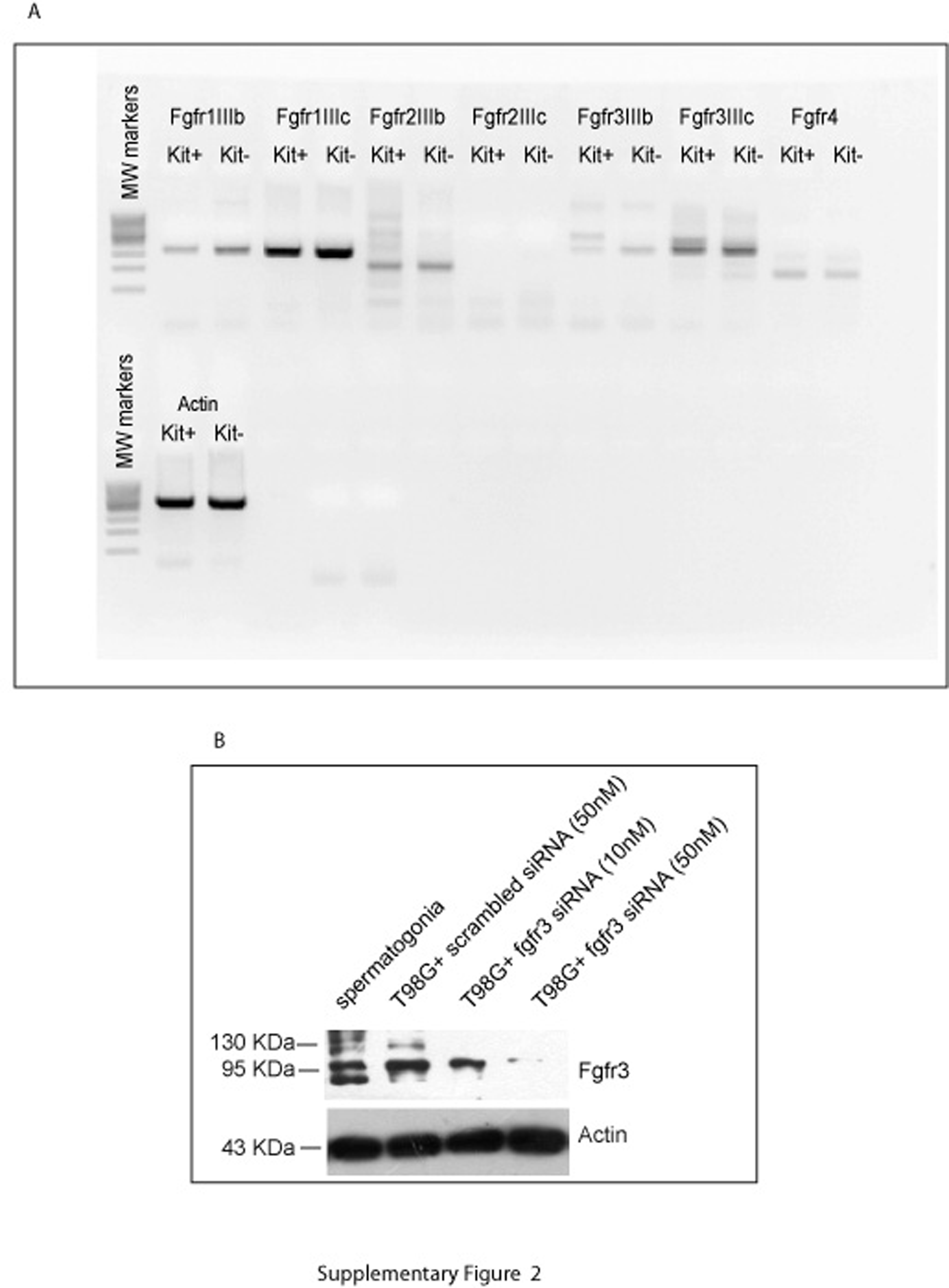

Supplement: Supplementary Figure 2 [file cddis201556x2.tif]

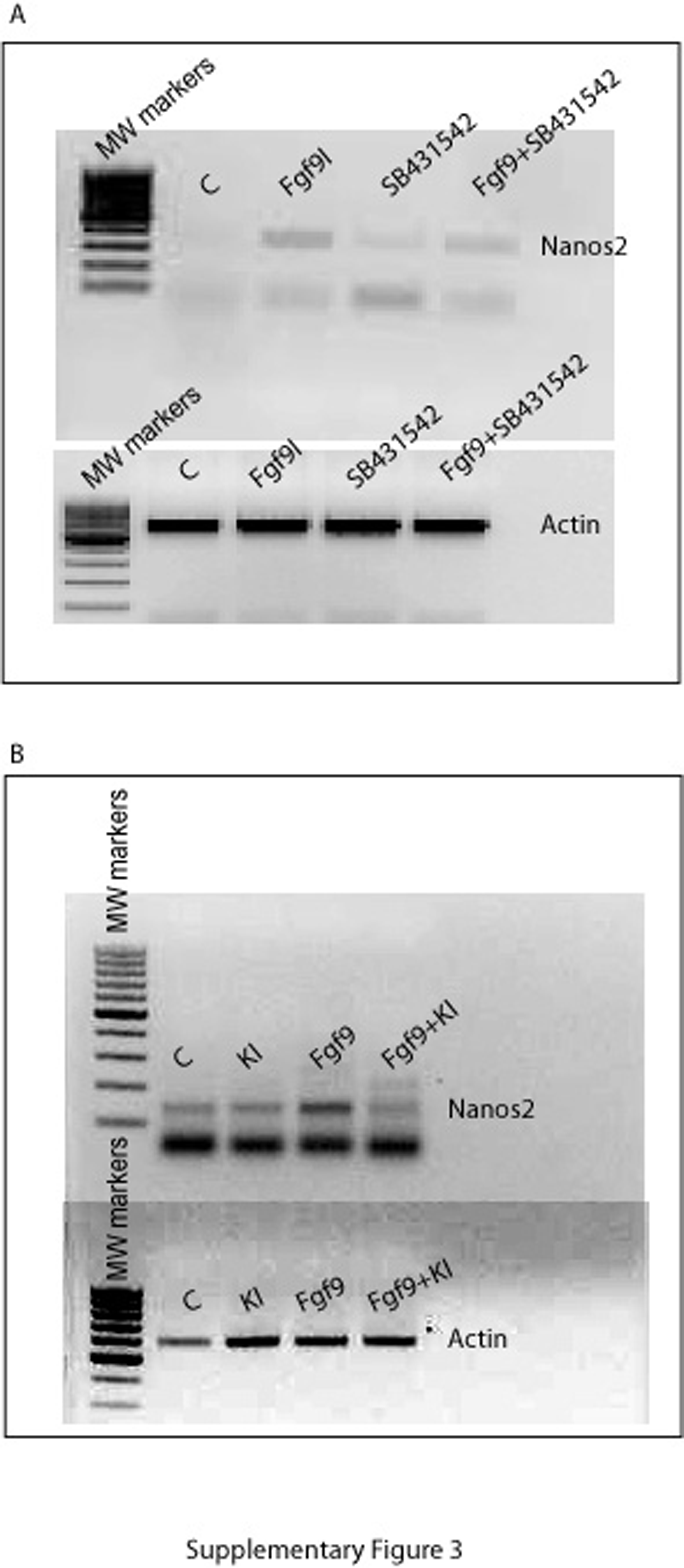

Supplement: Supplementary Figure 3 [file cddis201556x3.tif]
