# Supplementary material for: Fgf9 inhibition of meiotic differentiation in spermatogonia is mediated by Erk-dependent activation of Nodal-Smad2/3 signaling and is antagonized by Kit Ligand
Source: Cell Death Dis. 2015 Mar 12;6(3):e1688–. doi: 10.1038/cddis.2015.56 (PMC4385934; doi:10.1038/cddis.2015.56)
Supplement: Supplementary Figure Legends [file cddis201556x4.doc]

**Supplementary Figure 1**

A: EGFP fluorescence in immunomagnetic purified Kit positive spermatogonia from transgenic mice in which reporter gene expression is driven by Kit transcription regulatory sequences (ref. 16).

B: Kit immunostaining in immunomagnetic purified Kit positive spermatogonia from transgenic mice in which EGFP expression is driven by Kit transcription regulatory sequences. Purity of the Kit+ population was estimated to be over 95%.

**Supplementary Figure 2**

A: Full view of the gel utilized for the experiment shown in Figure 1A (representative semiquantitative RT-PCR analysis of mRNA expression of different Fgfr isoforms in Kit+ and Kit- spermatogonia).

B: RNrference for FGFR3 expression in the T98G human glioblastoma cell line. 48 hr after siRNA transfection at the indicated concentrations, cell extracts were subjected to western blot analysis using the anti-Fgfr3 antibody. In the first lane, protein extracts from mouse primary spermatogonia were run as a control. In both cell types the size of the major Fgf3r/FGFR3 band is approximately 95 KDa; a minor specific band of approximately 120 KDa was also detected.

**Supplementary Figure 3**

A: Full view of the gel utilized for the experiment shown in Figure 6A-left panel (semiquantitative RT-PCR analysis of mRNA expression of Nanos2 in untreated Kit+ spermatogonia and in the same cells cultured overnight with Fgf9, in the presence or absence of the Alk4/7 selective inhibitor SB431542. In this experiment we used Nanos2 primers “1” listed in Suppl. Table 1).

B: Full view of the gel utilized for the experiment shown in Figure 6A-right panel (semiquantitative RT-PCR analysis of mRNA expression of Nanos2 in untreated Kit+ spermatogonia and in the same cells cultured overnight with Kl, with Fgf9, or with both growth factors. In this experiment we used Nanos2 primers “2” listed in Suppl. Table 1).
