# Supplementary material for: Fgf9 inhibition of meiotic differentiation in spermatogonia is mediated by Erk-dependent activation of Nodal-Smad2/3 signaling and is antagonized by Kit Ligand
Source: Cell Death Dis. 2015 Mar 12;6(3):e1688–. doi: 10.1038/cddis.2015.56 (PMC4385934; doi:10.1038/cddis.2015.56)
Supplement: Supplementary Table 1 [file cddis201556x5.doc]

Supplementary Table 1.

A. Primers used for RT-PCR experiments

| **Gene** | **Forward (5′–3′)** | **Reverse (5′–3′)** |
| --- | --- | --- |
| *Cripto* | CACCTGGCTGCCCAAGAAGTGTTCC | GCCAGCTAGCATAAAAGTGGT |
| *Nodal* | GCCAAGAAGAGGATCTGGTA | CTGTCTGGCAAATGATGTCG |
| *Fgfr1IIIb* | CTTGACGTCGTGGAACGATCT | CACGCAGACTGGTTAGCTTCAC |
| *Fgfr1IIIc* | CTTGACGTCGTGGAACGATCT | AGAACGGTCAACCATGCAGAG |
| *Fgfr2IIIb* | CCCATCCTCCAAGCTGGACTGCCT | CAGAGCCAGCACTTCTGCATTG |
| *Fgfr2IIIc* | CCCATCCTCCAAGCTGGACTGCCT | CAGAACTGTCAACAATGCAGAGTG |
| *Fgfr3IIIb* | CAAGTTTGGCAGCATCCGGCAGA | TCTCAGCCACGCCTATGAAATTGGTG |
| *Fgfr3IIIc* | CAAGTTTGGCAGCATCCGGCAGA | CACCACCAGCCACGCAGAGTGATG |
| *Fgfr4* | TTCTGTTCCAGCCTTATGCCCC | TGATGCCCCTTTCACCAAGATG |
| *Lefty1* | CAGGCAAGAGGTTCAGCCAGAA | CGCTGCTCCATTCCGAACACTA |
| *Lefty2* | ACACGCTGGACCTCAAGGACTA | GTACATCTCCTGGCGACAGCAT |
| *ActRIIB* | GGAAGGCTCAGCTCAATGAACGA | TGCCGGGTGTGCTGAAGATTTC |
| *Alk4* | AGAGGGTGGGGACCAAAC | TGCTTCATGTTGATTGTCTCG |
| *Alk7* | ACACTGCACCTTCCCACAG | AATTGTCCTTGCGATTGTTCTT |
| *Nanos2 (1)* | ACAGCAGTCAGTCAGTCTC | CCGAGAAGTCATCACCAG |
| *Nanos2*  *(2)* | CTGGATGTCTGCCTACCATA | CTCCACATAGAGGACACACA |
| *Actin* | GACATGGAGAAGATCTGGCA | GTTTCATGGTGCCACAGGA |

B. Primers used for RT-qPCR experiments

| **Gene** | **Forward (5′–3′)** | **Reverse (5′–3′)** |
| --- | --- | --- |
| *Cripto* | CACCTGGCTGCCCAAGAAGTGTTCC | GCCAGCTAGCATAAAAGTGGT |
| *Nodal* | AGCAGAAAAGTGTTGGCATCAG | CTGTCTGGCAAATGATGTCG |
| *Gapdh* | AACTTTGGCATTGTGGAAGG | CACATTGGGGGTAGGACAC |
| *Fgfr1IIIb* | CTTGACGTCGTGGAACGATCT | TACTCCCGTTCACCTCGATG |
| *Fgfr1IIIc* | CTTGACGTCGTGGAACGATCT | TTCACCTCGATGTGCTTCAG |
| *Fgfr2IIIb* | CCCATCCTCCAAGCTGGACTGCCT | TTTATCCCCGAGTGCTTCAG |
| *Fgfr2IIIc* | CCCATCCTCCAAGCTGGACTGCCT | CCTTCAGGACCTTGAGGTAGG |
| *Fgfr3IIIb* | CAAGTTTGGCAGCATCCGGCAGA | GGATGTGTGGCTGTGCATCG |
| *Fgfr3IIIc* | CAAGTTTGGCAGCATCCGGCAGA | GCATCGCTGTACACCTTGCA |
| *Fgfr4* | CGGGACTAGCTGCAAAACTT | TGATGCCCCTTTCACCAAGATG |
