# Supplementary material for: Fgf9 inhibition of meiotic differentiation in spermatogonia is mediated by Erk-dependent activation of Nodal-Smad2/3 signaling and is antagonized by Kit Ligand
Source: Cell Death Dis. 2015 Mar 12;6(3):e1688–. doi: 10.1038/cddis.2015.56 (PMC4385934; doi:10.1038/cddis.2015.56)
Supplement: Supplementary Table 2 [file cddis201556x6.doc]

Supplementary Table 2

| **Peptide/protein target** | **Antigen sequence (if known)** | **Name of Antibody** | **Manufacturer, catalog #, and/or name of individual providing the antibody** | **Species raised in; monoclonal or polyclonal** | **Dilution used** |
| --- | --- | --- | --- | --- | --- |
| FGFR3 |  | Anti-FGFR3 | Santacruz SC-123 | rabbit polyclonal | 500 |
| Actin |  | anti-Actin | Sigma A2066 | rabbit polyclonal | 2000 |
| Smad2 |  | anti-Smad2 | GeneTex GTX111131 | rabbit polyclonal | 500 |
| pSmad2 |  | anti-phosho-Smad2 Ser465/467 | Cell signaling 138D4 | rabbit monoclonal | 500 |
| Cripto |  | anti-Cripto | Abcam Ab139725 | rabbit polyclonal | 500 |
| SCP3 |  | anti-SCP3 | Santacruz SC-74569 | mouse monoclonal | 1000 |
| Stra8 |  | anti-Stra8 | Abcam Ab49405 | rabbit polyclonal | 1000 |
| Tubulin |  | anti-Tubulin | Sigma T9026 | mouse monoclonal | 2000 |
| pAkt |  | anti p-Akt (Ser 473) | SantaCruz SC-7985 | rabbit polyclonal | 1000 |
| pErks |  | anti p-Erks (Thr202/Tyr204) | Cell Signaling 9101S | rabbit polyclonal | 1000 |
| Akt |  | anti Akt | SantaCruz, SC-5298 | mouse monoclonal | 1000 |
| Kit | see reference 26 | anti-Kit | Proteogenix | rabbit polyclonal | 1000 |
| Kit |  | CD117-PE | Miltenyi Biotech  130-102-795 | mouse monoclonal | 10 |
| Erk2 |  | Anti-Erk2 | SantaCruz, SC-154 | rabbit polyclonal | 1000 |
